# Effect of “finite pool of worry” and COVID-19 on UK climate change perceptions

**DOI:** 10.1073/pnas.2018936118

**Published:** 2021-01-04

**Authors:** Darrick Evensen, Lorraine Whitmarsh, Phil Bartie, Patrick Devine-Wright, Jennifer Dickie, Adam Varley, Stacia Ryder, Adam Mayer

**Affiliations:** ^a^Department of Politics and International Relations, University of Edinburgh, Edinburgh EH8 9LF, United Kingdom;; ^b^Department of Psychology, University of Bath, Bath BA2 7AY, United Kingdom;; ^c^Department of Computer Science, Heriot-Watt University, Edinburgh, Currie EH14 4AL, United Kingdom;; ^d^Department of Geography, University of Exeter, Exeter EX4 4RJ, United Kingdom;; ^e^Department of Biological and Environmental Sciences, University of Stirling, Stirling FK9 4LA, United Kingdom;; ^f^Center for Earth Observations and Global Change, Michigan State University, East Lansing, MI 48823

**Keywords:** climate change, finite pool of worry, COVID-19, longitudinal, United Kingdom

## Abstract

Research reveals that a “finite pool of worry” constrains concern about and action on climate change. Nevertheless, a longitudinal panel survey of 1,858 UK residents, surveyed in April 2019 and June 2020, reveals little evidence for diminishing climate change concern during the COVID-19 pandemic. Further, the sample identifies climate change as a bigger threat than COVID-19. The findings suggest climate change has become an intransigent concern within UK public consciousness.

The “finite pool of worry” hypothesis states that environmental and climate concerns diminish as other worries rise in prominence (1, 2). Originally developed to explain fluctuating environmental concern broadly (3), it has since been applied to attitudes toward and action on climate change holistically (4–7). Whitmarsh (8) and Weber (9) reveal effects of the 2008 economic recession on perceptions of climate change—displacing climate change concern, “expressed through doubt about the reality or severity of the issue” (8).

If the “finite pool of worry” hypothesis were still a robust explanation for concern about climate change, one might expect health and economic concerns associated with COVID-19 to reduce perceptions of climate change severity or reality. Nevertheless, the April 2020 Climate Change in the American Mind survey found a relative lack of evidence supporting a “finite pool of worry” (10). Further, UK data reveal increases from May 2019 to May 2020 in perceived urgency of climate change and support for climate change mitigation policies—the opposite of what “finite pool of worry” would predict (11). Both 2020 surveys were based on relatively small sample sizes (United States *n* = 312, United Kingdom *n* = 284), and their comparison with previous surveys was via a repeated cross-sectional design—different people responded to each survey. To assess the robustness of the emerging findings, we conducted a survey with a large-scale representative and longitudinal sample (*n* = 1,858) in the United Kingdom—identical respondents in April 2019 and June 2020.

## Results

Beliefs about the extent to which climate change is real and anthropogenically induced were measured 14 months apart, as were beliefs about the seriousness of climate change. Of our four measures of seriousness, three showed no significant difference between April 2019 and June 2020 (paired-samples *t* tests)—only seriousness of climate change for “you and your family” varied (slight decrease in perceived severity of 0.10 on a 5-point scale). Nevertheless, the effect size (Cohen’s d) was very small (0.09).

Of the five beliefs about climate change reality, four showed significant differences at *P* < 0.05 (Table 1), but the Cohen’s d (strength of the relationship) in each case was minimal, below the 0.10 threshold for what is considered small (12), suggesting minor differences were only made significant by the large sample size. In each significant relationship there was slightly more agreement in June 2020 that climate change was real and anthropogenically induced.

**Table 1.** Change in beliefs about climate change from 2019 to 2020

Item	Mean difference (T2 –T1)	Significance	Cohen’s d (effect size)
I am convinced that climate change is really happening	0.07	0.029	0.08
Claims that human activities are changing the climate are exaggerated	−0.13	0.001	0.08
The evidence for climate change is unreliable	−0.11	0.002	0.05
Climate change is just a natural fluctuation in Earth’s temperatures	−0.11	0.001	0.07
The media is often too alarmist about issues like climate change	−0.05	0.149	0.03

Beyond the differences over time in our longitudinal panel, the survey respondents specifically acknowledged the continued relevance of climate change in the era of COVID-19. When asked, “Which do you consider to be a bigger threat to the future in… (1) the UK (2), Europe, and (3) the world?” COVID-19 was perceived marginally as a bigger threat for the United Kingdom (43% vs. 42%). Nevertheless, COVID-19 was perceived as less of a threat than climate change by the plurality of the sample for Europe (40% vs. 45%) and the world (33% vs. 55%) (unaccounted-for percentages responded “neither” or “don’t know”). This survey was conducted at a time when all four nations of the United Kingdom were still in a relatively strict lockdown, and had been for 3 months.

Leading variables affecting propensity to state that climate change was a bigger threat to the future than COVID-19 were perceived climate change seriousness (average of four items); climate change being real (average of five items); support for declaration of a climate emergency by the UK, Scottish, and Welsh governments; being male; and liberal political orientation (Table 2).

**Table 2.** Predictors of climate change as a bigger perceived threat than COVID-19 (binary logistic regressions)

Variable	Odds ratio, threat to United Kingdom	Odds ratio, threat to Europe	Odds ratio, threat to the world
Climate change seriousness	1.68	1.66	1.42
Climate change is real	1.49	1.55	1.52
Support “climate emergency”’ declaration	1.33	1.31	1.40
Male (vs. female)	1.56	1.50	1.35
Very conservative to very liberal (7-point scale)	1.13	1.20	1.24

Statistical significance for the three climate change attitude/belief variables was *P* < 0.001 in all models; the two sociodemographic variables had *P* < 0.05 in all models. Model pseudo-R^2^ values across the three models were Nagelkerke = 0.34 to 0.36 and Cox and Snell = 0.26 to 0.27.

**Fig. 1.**
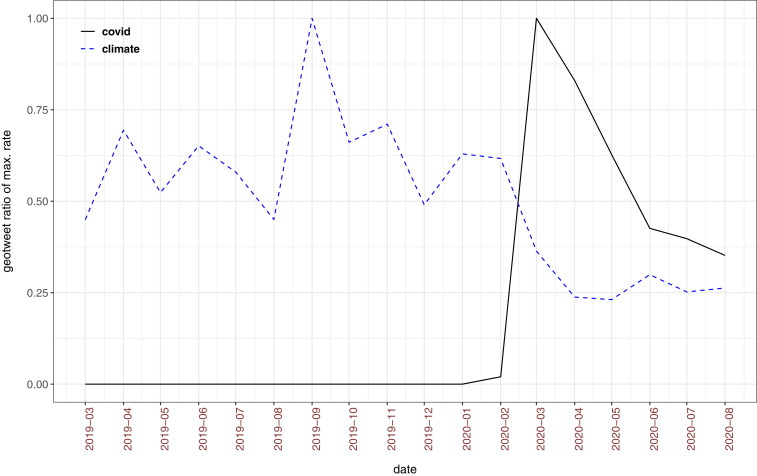
Twitter attention to “climate” and “covid,” March 2019 to August 2020. Data are based on geotweets in the UK region; *y* axis depicts the ratio of the maximum rate per month—1.00 is the month with the highest percentage of tweets about a term (either “covid” or “climate”).

To further explore the extent to which attention to climate change evolved since COVID-19, Twitter data were collected, specifically tweets with a geolocation attribute identified as within the UK region, from 1 March 2019 through 17 August 2020 (∼124 million tweets). This dataset reveals that the percentage of attention to climate change compared to all other topics on this social media platform decreased when COVID-19 emerged in discourse. From March 2019 through February 2020, tweets including the term “climate” comprised 0.145% of all UK geolocated tweets (ranging from 0.104 to 0.232% on a monthly basis), but in the period March to August 2020 “climate” tweets comprised 0.064% of the geolocated tweets (from 0.054 to 0.085% monthly). In comparison, tweets including “covid” comprised 1.511% of UK-geolocated tweets during March to August 2020 (Fig. 1).

## Discussion

Our results in aggregate offer very little evidence to support the hypothesis that a “finite pool of worry” is diminishing concern about climate change. The 14 months from the first survey to the second survey included the endless barrage of exposure, both direct and through mass/social media, to the effects of coronavirus, but they also included mass protests by the youth climate strikes and Extinction Rebellion, prominent news of Australian wildfires and melting glaciers in Europe, and UK government action on climate change through a legal commitment to net-zero carbon emissions by 2050 and creation of a UK Climate Assembly bringing together UK citizens to make recommendations for future action.

Perhaps the weight of such actions that could increase perceived severity and reality of climate change balanced against the effects of COVID-19 encroaching upon respondents’ finite pool of worry. Nevertheless, the availability heuristic (13) and the extreme psychological proximity of COVID-19 (14) would suggest that COVID-19 should affect the UK respondents much more substantially than socially distant events that occurred in 2019 (e.g., only 3% of our sample reported themselves or a family member engaging in youth climate strikes, 3% for Extinction Rebellion protests, and 1% for the UK Climate Assembly). The Twitter data suggest that although attention to climate change seems to have waned during the pandemic, this does not directly translate into altered perceptions of severity or reality of climate change.

While the findings certainly do not refute the existence of people having a finite pool of worry, they might instead suggest that over the decade since some of the previous research in this area was conducted (8, 9) climate change may have increasingly become a “permanent member” of more people’s pool of worry, at least in the United Kingdom. Indeed, increased stability of attitudes and beliefs as people gain more familiarity with a topic has long been recognized (15, 16).

The intransigence of our sample’s beliefs about climate change’s severity and reality could be positive or negative for the realization of concerted action on climate change. That perhaps the biggest news story and global crisis of most of our lifetimes, with enormous health and economic repercussions, is not associated with a decrease in perceived severity of climate change is good news. A separate question of high policy import is what events or communication would increase worry, and the extent to which that worry would motivate concrete action.

We asked all respondents if they had read or heard about the following in the last year: youth climate strikes (“Fridays for Future”), Extinction Rebellion protests, Australian wildfires, storms and flooding in the United Kingdom, melting glaciers in the Alps and Greenland, and the UK Climate Assembly. None of these had significant correlations (at *P* < 0.05) with respondents’ change in perceived climate change severity from April 2019 to June 2020. This suggests climate change stories are not effecting change in perceived severity. This cautions against being overly optimistic about the capacity for climate change news and risk communication to heighten issue concern. Again, however, COVID-19 clearly could be a confounding historical event here.

Our findings suggest the “finite pool of worry” hypothesis in relation to climate change requires more attention, and potentially nuanced revision. Future research that examines climate beliefs, attitudes, and affect over periods of high societal attention to climate change with few major conflicting crises could shed light on the relationship, as could additional research that examines additional operationalizations of “worry” to include measures based more on affect. Repeated cross-sectional survey data suggest a similar pattern to ours (10, 11); does this ambiguity over finite pool of worry extend to other national and cultural contexts? Finally, does the obduracy of beliefs about climate change work both ways—will perceived severity and reality of climate change increase in the absence of major COVID-19 concerns, or does a strongly reified set of beliefs mean little change can be expected in either direction?

## Materials and Methods

The longitudinal online survey was first conducted 8 to 12 April 2019 with 2,777 UK respondents, constrained with quotas to represent the UK population on age, sex, UK census region of residence, social grade, education, party vote in the 2017 general election, vote in the 2016 EU (Brexit) referendum, and attention paid to politics. The follow-up survey, 16 to 30 June 2020, attracted 1,858 respondents (67% from 2019).

The core questions replicated across both surveys, allowing for us to compare evolution in perspectives on climate change, were a four-item scale on beliefs toward seriousness of climate change (one factor, 82% of variance explained, lowest loading at 0.82, and reliability alpha of 0.93) and a five-item scale on beliefs about the extent to which climate change is real (one factor, 68% variance explained, lowest loading 0.64, and reliability alpha 0.88). These items were replicated from reliable and valid measures used in prior research (17); see *SI Appendix* for exact items.

Our Twitter data come from a dataset of almost 124 million geolocated UK tweets analyzed using the Twitter Streaming API (application programming interface) from 1 March 2019 through 17 August 2020. While this API is known to filter the data stream, it does not impose a strong filter on tweets with a spatial attribute due to their relatively low numbers; therefore, our database contains the vast majority of geolocated tweets sent in the United Kingdom during the time period.

Human subjects’ approval was granted by the University of Edinburgh’s Social and Political Science ethics committee and the University of Exeter’s Geography ethics committee. All survey respondents were asked for consent to participate at the start of the survey and allowed to withdraw at any time. Data collection was anonymous; no identifying information was collected.

## Supplementary Material

Supplementary File

## Data Availability

Some study data are available.
