# Supplementary material for: Effect of “finite pool of worry” and COVID-19 on UK climate change perceptions
Source: Proc Natl Acad Sci U S A. 2021 Jan 4;118(3):e2018936118. doi: 10.1073/pnas.2018936118 (PMC7826411; doi:10.1073/pnas.2018936118)
Supplement: Supplementary File [file pnas.2018936118.sapp.pdf]

PNAS
www.pnas.org

Supplementary Information for
Effect of 'finite pool of worry' and COVID-19 on UK climate change perceptions

Darrick Evensen*, Politics and International Relations, University of Edinburgh, UK
Lorraine Whitmarsh, Psychology, University of Bath, UK
Phil Bartie, Computer Science, Heriot-Watt University, UK
Patrick Devine-Wright, Geography, University of Exeter, UK
Jennifer Dickie, Biological and Environmental Sciences, University of Stirling, UK
Adam Varley, Biological and Environmental Sciences, University of Stirling, UK
Stacia Ryder, Geography, University of Exeter, UK
Adam Mayer, Center for Earth Observations and Global Change, Michigan State University, USA

*Corresponding author: Darrick Evensen
Email: darrick.evensen@ed.ac.uk

This PDF file includes:

Supplementary text

Other supplementary materials for this manuscript include the following:

None

Supplementary Information Text

Methods

Response rates are not indicative when using online quota sampling, as non-response cannot be easily defined; demographic approximation of population values should be consulted. In our June 2020 survey, only one quota variable differed noticeably – age (when weighting the sample by age, however, no differences in the relationships above were found). The June 2020 sample was slightly older (median: 56 years, compared to 48 years in April 2019).

The full data set is available from the authors. The survey was administered by YouGov, an online survey panel provider. Data collection and data handling complied with EU General Data Protection Regulation (GDPR) laws.

Items on climate change seriousness and reality included in the survey:

1. How serious a threat, if at all, do you think climate change is to each of the following? [response options: Not at all serious, Not very serious, Fairly serious, Very serious, Extremely serious, Don't know]
   - You and your family
   - The UK as a whole
   - People in developing countries
   - Wildlife and ecosystems

2. To what extent do you agree or disagree with the following statements? [response options: Strongly disagree, Moderately disagree, Slightly disagree, Slightly agree, Moderately agree, Strongly agree, Don't know]
   - Claims that human activities are changing the climate are exaggerated
   - I am convinced that climate change is really happening
   - The evidence for climate change is unreliable
   - Climate change is just a natural fluctuation in the earth's temperatures
   - The media is often too alarmist about issues like climate change
